# Striding Toward Malaria Elimination in China

**DOI:** 10.4269/ajtmh.15-0391

**Published:** 2015-08-05

**Authors:** Michelle S. Hsiang, Roly D. Gosling

**Affiliations:** Department of Pediatrics, University of Texas Southwestern Medical Center, Dallas, Texas; Malaria Elimination Initiative, Global Health Group, University of California, San Francisco, California; Department of Pediatrics, UCSF Benioff Children’s Hospital, University of California, San Francisco, California

In this month’s *American Journal of Tropical Medicine and Hygiene*, China’s remarkable progress in malaria control and elimination over the last decade is showcased in two articles. Feng and others reported annual national trends from 38,972 reported cases in 2004, to a peak of 60,193 (or 0.40/10,000) in 2006, to just 4,128 in 2013.^1^ Chen and others reported similar data from Zhejiang province, whereby reported malaria cases peaked in 2007 at 598 (0.12/10,000)^2^ and then declined to 215 in 2014.^3^ As the populations in many of China’s provinces exceed those of most other countries—the population of Zhejiang was 55 million in 2013—province level experiences and achievements are worth noting.

There are 31 provinces, municipalities, or autonomous regions in China. In 2010, when the national elimination strategy was formulated, 19 were considered to have endemic malaria, defined as the presence of confirmed local case(s) in the prior 3 years. The elimination strategy set forth goals to eliminate local transmission from all areas but the Yunnan–Myanmar border by 2015 and to be completely free of local malaria transmission by 2020.^4^ The abovementioned two articles suggest that prospects for meeting the 2015 goal are good. From 2004 to 2013, the proportion of cases nationwide representing local transmission declined from 84% to 2%, or less than 100 cases. In Zhejiang, the proportion of locally transmitted cases has declined from roughly 15% in 2005 to none in 2012, with the lack of local transmission sustained as of 2014. Beyond Zhejiang, local transmission in China remains only in foci along the Yunnan–Myanmar border (*Plasmodium falciparum* and *P*. *vivax*) and in Motuo County in the Tibetan Autonomous Region (*P*. *vivax* only).

This year, the World Health Assembly agreed to a global malaria strategy that aims to eliminate malaria in 35 new countries by 2030.^5^ At the 2014 East Asia Summit, heads of state agreed to the goal of an Asia Pacific free of malaria by this same year.^6^ As countries pursue these goals, it will be important to understand how China got to where it is today and to recognize the challenges that lie ahead. Beyond obvious socioeconomic development and urbanization, several key enabling factors should be highlighted.

First, the importance of political and financial commitment for the final push toward malaria elimination cannot be underestimated. When malaria comes under control, there is a tendency for commitment and resources to wane in the face of competing priorities. However, the Chinese government has recognized broader purposes, that is, to promote not only health, but also economic and social development, and to align with the global malaria elimination agenda.^7^ There were mandates for legislation and coordinated action across several ministries and commissions (including Finance, Science and Technology, Entry-Exit Inspection and Quarantine, and General Logistics) and governments at all levels. The government’s commitment is best manifested by continued strong domestic financial support despite China losing eligibility for new funding from the Global Fund to Fight AIDS, Tuberculosis and Malaria in 2012.

Second, as the epidemiology of malaria is heterogeneous, particularly in low-transmission settings, China has stratified its approach to malaria elimination. Counties are classified into four categories: Type 1, confirmed local cases in the past three consecutive years, with incidence ≥ 1/10,000 in at least 1 year; Type 2, confirmed local cases in the past 3 years and annual incidence < 1/10,000; Type 3, no local cases for at least 3 years, and thus only imported cases; and Type 4, no history of any local cases, and thus only imported cases. Within counties, further stratification occurs to the level of villages, facilitating targeting and tailoring of interventions to local conditions. For example, vector control would be more of a priority in areas with ongoing local transmission whereas activities to address imported malaria would be would more of a priority in Type 3 and 4 areas. Re-stratification is ongoing to ensure that intervention strategies are dynamic and appropriate.

Third, passive and active surveillance programs have been strengthened.^8^ On a national level, the proportion of reported cases based on only clinical diagnosis, without laboratory confirmation, was > 30% on average over the study period, but dropped to only 1% in 2013. In Zhejiang, the median time from illness onset to laboratory confirmation of malaria declined from a median of 5 to 3 days over the study period. Importantly, in 2004, in response to the severe acute respiratory syndrome (SARS) outbreak, a national web-based system was established for reporting of 37 notifiable conditions at the level of the township.^9^ This system provides China with real-time malaria data. A “1-3-7” strategy was developed to link passive with active surveillance and response, with case reporting within 1 day, case investigation within 3 days, and focused investigation and action within 7 days.^10^

Finally, a key strategy has been the use of mass drug administration (MDA) for malaria control and elimination. During the 1950s and 1960s, China implemented MDA campaigns on a large scale, treating tens of millions of people for control of *P*. *falciparum* and *P*. *vivax* in high-transmission settings. More recently, MDA has been reserved for targeted use in smaller populations. Specifically, high-risk individuals receive primaquine before the transmission season as “spring treatment” to eliminate any potential *P*. *vivax* liver stage parasites, and drugs are administered during the transmission season as chemoprophylaxis.^11^–^14^ In 2006, when there was a resurgence of *P*. *vivax* in Anhui and cases there accounted for 58% of China’s indigenous cases, MDA was critical to local control, and also likely resulted in fewer imported cases to nearby Zhejiang. MDA for malaria is not currently recommended by the World Health Organization, but new policy is being considered.^15^

Moving forward, what is next for China? With the shift from control to elimination, new challenges have arisen.^16^,^17^ The two articles published in this issue clearly show that malaria is increasingly imported, a disease of adult men, and occurs throughout the year, rather than in traditional peaks associated with rainy seasons. Maintaining constant vigilance regarding imported malaria and prevention of reintroduction will be important. New strategies to target high-risk individuals, such as Chinese businessmen and laborers working in Africa, will need to be designed and implemented with private companies. The question of whether the ambitious task of border screening is feasible or effective will need to be tested. Collaboration with neighbors near and far, such as through the Asia Pacific Malaria Elimination Network, will be critical. Will the world’s most populous and fastest developing country prevail in its goal for malaria elimination? Likely yes. The next question will be whether China can take these lessons learned, and contribute leadership, expertise, and resources to help realize worldwide eradication, a goal recently set for 2040.^18^
